# Effects of *Chlorella vulgaris* Enhancement on Endogenous Microbial Degradation of Marine Oil Spills and Community Diversity

**DOI:** 10.3390/microorganisms10050905

**Published:** 2022-04-26

**Authors:** Zhao Song, Mei Liu, Bo Bao, Jian Guo, Hengcong Tao, Baikang Zhu, Qingguo Chen

**Affiliations:** 1 Zhejiang Provincial Key Laboratory of Petrochemical Pollution Control, Zhejiang Ocean University, Zhoushan 316022, China; s20070700038@zjou.edu.cn (Z.S.); liumei@zjou.edu.cn (M.L.); hengcongtao@zjou.edu.cn (H.T.); 2 School of Marine Science & Technology, Zhejiang Ocean University, Zhoushan 316022, China; s6121z@163.com

**Keywords:** *Chlorella vulgaris*, endogenous microorganism, enhanced degradation, marine oil spill

## Abstract

Biofortification could improve the bioremediation efficiency of microbes in the reparation of marine environmental damage caused by oil spills. In this paper, *Chlorella vulgaris* LH-1 was used as a fortifier to enhance the degradation of a marine oil spill by endogenous microorganisms. The addition of *C. vulgaris* LH-1 increased the degradation efficiency of crude oil by 11.09–42.41% and considerably accelerated oil degradation efficiency. Adding *C. vulgaris* LH-1 to a crude oil environment can improve the activity of endogenous seawater microorganisms. The results of high-throughput sequencing showed that the main bacterial genera were *Oceanicola*, *Roseibacillus*, and *Rhodovulum* when exotrophic *C. vulgaris* LH-1 and seawater endogenous microorganisms degraded low-concentration crude oil together. However, the addition of high-concentration nutrient salts changed the main bacterial genera in seawater to unclassified *Microbacterium*, *Erythrobacter*, and *Phaeodactylibacter*. The addition of *C. vulgaris* LH-1 increased the abundance of marine bacteria, *Rhodococcus*, and *Methylophaga* and decreased the abundance of *Pseudomonas*, *Cladosporium*, and *Aspergillus*. The functional prediction results of phylogenetic investigation of communities by reconstruction of unobserved states indicated that *C. vulgaris* LH-1 could improve the metabolic ability of seawater endogenous microorganisms to degrade endogenous bacteria and fungi in crude oil.

## 1. Introduction

With the rapid growth of the global economy, worldwide demand for oil, as well as the occurrence of oil spill accidents in offshore oil facilities, such as tankers and offshore oil platforms, has increased greatly [1,2,3]. Oil spill accidents cause a devastating blow to the marine environment [4,5,6,7]. Volatile organic compounds (VOCs) in crude oil are harmful to humans [8,9,10].

Bioremediation as a reparation method for oil spills has advantages for marine bacteria, fungi, and algae in the environment because of its ability to remove hydrocarbon pollutants [11,12,13]. This method permeates the oil for repair and is commonly used as a microbial remediation strategy [10]. This method includes microorganism, biological stimulation, and the combination of biological stimulation and biological strengthening. The degradation of petroleum hydrocarbons by microorganisms is stimulated by the selection of microorganisms and surrounding nutrients. Therefore, much research has focused on the metabolic properties of various types of bacteria, fungi, and microalgae to repair oil-contaminated areas; the addition of exogenous microbes to enhance degradation of pollutants in the environment; and biofortification, which increases the concentration of microorganisms with a particular metabolic capacity [14,15]. Degradation ability can be improved by changing the environment of bacterial species [16] and modifying nutrients, such as nitrogen and phosphorus, as a biological stimulant to improve the biodegradation rate of crude oil in oil-contaminated seawater [17,18]. Some scholars co-cultured exogenous and endogenous bacteria for crude oil degradation and achieved a considerably better degradation efficiency than the inoculation of endogenous bacteria only [19]. The co-culture of local flora and exogenous fungi for crude oil degradation also considerably enhanced the activity of endogenous microorganisms and increased the uniformity and diversity of endogenous bacteria [13].

*Chlorella vulgaris* is a typical green microalga and has been used in the treatment of nitrogen and phosphorus pollution in water environments in recent years, owing to its low nutritional requirements and fast growth cycle [20]. It is an organism that can effectively degrade hydrocarbons [21]. However, as far as we know, few studies have reported on the microalgal enhancement of the endogenous microbial degradation of oil spills.

The purpose of this study was to investigate the effects of *C. vulgaris* on the degradation of crude oil with different concentrations by endogenous bacteria and fungi and investigate the composition of endogenous bacterial and fungal communities after *C. vulgaris* enhancement using high-throughput sequencing.

## 2. Materials and Methods

### 2.1. Crude Oil and Chemicals

The crude oil sample, *C. vulgaris* LH-1, and coastal seawater used for cultivating microbes were the same as those described by Li et al. [11]. The crude oil sample used in this study was from China Xingzhong Zhonghua Petroleum Transportation (Zhoushan) Co., Ltd., with a density of about 0.91 g·cm^−3^. Seawater used for cultivating microbes was collected from coastal waters of Zhoushan (29°57′ N, 122°11′ E). The microalga *C. vulgaris* LH-1 used in this study was collected from coastal waters of Zhoushan (Genbank accession number KU302752). The chemical reagents used in this research were procured from China National Pharmaceutical Group.

### 2.2. Preparation of Inoculum and Experimental Medium

The inoculum containing endogenous bacteria and fungi was collected from coastal seawater in Zhoushan (29°57′ N, 122°11′ E) and filtered using a qualitative filter paper to reduce suspended solids. The inoculum was cryopreserved at 4 °C. The inoculant and experimental medium for microorganisms were the same as those described by Chen et al. [1]. *C. vulgaris* LH-1 was inoculated in the microalgal medium as described by Li et al. [11] at a ratio of 3 mL/100 mL and then cultured in a light incubator under 7000 LX light at 25 °C and shaken 2–3 times a day for 15 days. Afterward, the medium was removed by centrifugation at 5000 rpm at 4 °C for 10 min. The algae were retained, and purified water was added until an absorbance of about 1.0 at 600 nm for microalgal inoculation. The pH of the cultures was adjusted to 8.0. All media were sterilized and prepared for inoculation.

### 2.3. Biodegradation of Petroleum Hydrocarbons

Three different groups were set: group A was inoculated with seawater microorganism, group B was inoculated with seawater microorganism and *C. vulgaris* LH-1, and group C was inoculated with *C. vulgaris* LH-1. Seawater inoculum and microalgal suspension were inoculated at 3 mL/1 mL (30 mL medium). A 3 mL of inoculum and 3 mL of algal fluid were added to a 100 mL conical flask with 30 mL of medium. All seven inoculated media were cultivated at 25 °C for 14 days in an oscillating incubator. Crude oil concentrations before and after biodegradation were determined at 420 nm by spectrophotometry to calculate the crude oil degradation efficiency as described in Chen et al. [1].

### 2.4. Biological Activity Assay

Fluorescein diacetate (FDA) was used to characterize the biological activity of microorganisms, which is correlated with biomass. FDA is hydrolyzed by enzymes produced by microorganisms to produce luciferin, which can be determined by spectrophotometry at 490 nm for the characterization of microbial bioactivity.

### 2.5. PCR Amplification and Illumina Miseq Sequencing

All seven experimental samples were cultivated for 14 days before high-throughput sequencing. The specific methods for microbial DNA extraction and purification were performed according to the protocols of Chen et al. [1]. The barcoded primers were prepared based on the method of Zhou et al. [22] and used to amplify specific regions using a thermonuclear polymerase chain reaction (PCR) system (Gene Amp 9700, ABI, USA). The PCR amplification procedure was in accordance with procedures described by Liu [23]. Sequencing was performed using an Illumina Miseq PE300 platform (Majorbio Bio-Pharm Technology Co., Ltd., Shanghai, China). Operational taxonomic units (OTUs) were clustered with 97% similarity cutoff using UPARSE (version 7.1 http://drive5.com/uparse/, accessed on 6 December 2019) with a novel greedy algorithm that performs chimera filtering and OTU clustering simultaneously. The taxonomy of each 16S rRNA gene sequence was analyzed using the RDP Classifier algorithm (http://rdp.cme.msu.edu/, accessed on 6 December 2019) against the Silva (SSU132) 16S rRNA and UNITE (8.0) internal transcribed spacer (ITS) database with a confidence threshold of 70%.

### 2.6. Statistical Analysis

UPARSE was used to perform the OTU clustering of sequences according to 97% similarity and remove single sequences and chimeras. RDP Classifier was used for the species classification annotation of each sequence. Bacterial 16S was compared with the Silva database (SSU132), and fungal ITS was compared using UNITE, with a comparison threshold of 70%.

Alpha diversity index was used to reflect the richness and diversity of the microbial community, and various diversity indices were analyzed statistically. Sobs, Chao, Ace, and other indices were used to represent the richness of the community. Shannon and Simpson indices were used to represent community diversity and species evenness, respectively. Coverage index was used to reflect the extent of community coverage. The differences in phyla and genera of each sample were analyzed by community column chart and community heat map.

Data analysis and variance analysis were performed using previously described methods [1,11] to deal with differences among parameters. All results are considered statistically significant at *p* < 0.05 [24].

## 3. Results

### 3.1. Crude Oil Biodegradation

**Figure 1:**
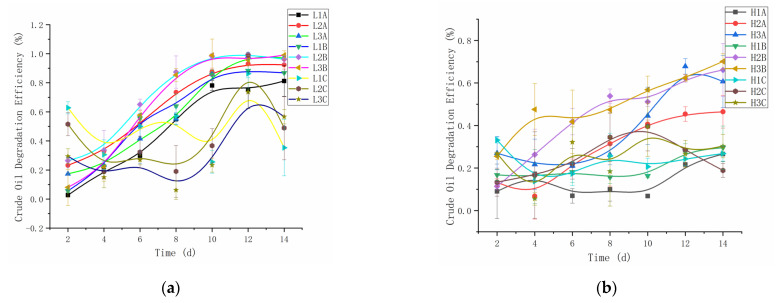
Biodegradation efficiency of crude oil treated with different nutrient concentrations within 14 days. (**a**) Seawater degradation efficiency of 0.01 g/L crude oil (**b**) Seawater degradation efficiency of 10.00 g/L crude oil.

Figure 1 shows the crude oil degradation efficiency after 14 days. A comparison of the degradation rates of crude oil under different treatments revealed the potential of endogenous microorganisms in crude oil degradation under the enhancement of *C. vulgaris* LH-1. The efficiency of *C. vulgaris* LH-1 alone in degrading crude oil, whether in low-concentration oil (35.36–56.68%) or high-concentration oil (18.87–25.34%), was lower than that of endogenous microorganisms or the mixture of bacteria and algae. The degradation efficiencies of L2B, L3A, and L3B on day 14 were 96.20%, 97.17%, and 99.05%, respectively, whereas the degradation efficiencies of L1A, L1B, and L2A were 81.20%, 86.85%, and 92.40%, respectively.

The trend of crude oil degradation efficiency conformed to the microorganism growth curve. The degradation was slow in the early stage, but the microorganism grew rapidly in the middle stage, and the degradation efficiency was accelerated. The degradation rate slowed down with the consumption of carbon sources and nutrients. At the same nutrient concentration, the degradation rate of crude oil with *C. vulgaris* LH-1 was higher than that without *C. vulgaris* LH-1. Crude oil contains a large number of toxic substances, such as polycyclic aromatic hydrocarbons (PAHs), and a higher crude oil concentration results in increased toxicity. Therefore, the normal growth of some seawater microorganisms is inhibited [25], and the growth period of microorganisms is also relatively prolonged, similarly to H2A, H3A. However, under the same nutritional conditions, H2B and H3B added to *C. vulgaris* LH-1 quickly entered the logarithmic growth phase, and the degradation rate was accelerated.

When *C. vulgaris* LH-1 is co-cultured with bacteria and fungi to degrade hydrocarbons, *C. vulgaris* LH-1 provides oxygen to the bacteria through photosynthesis and improve its degradation efficiency. Bacteria can also grow *C. vulgaris* LH-1 to provide nutrients. Without added nutrients, the degradation efficiency increased by 11.09% at the higher concentration of 10.00 g/L. The degradation efficiency increased by 42.41% after adding low-concentration nutrient salt and increased by 15.60% after adding high-concentration nutrient salt. The crude oil degradation efficiency enhanced by *C. vulgaris* LH-1 was remarkably improved by the introduction of exogenous *Chlorella*.

### 3.2. FDA Enzyme Activity

**Figure 2:**
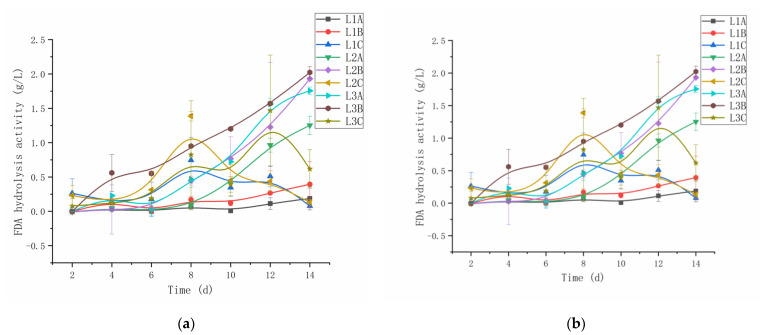
Hydrolytic activity under different treatments during biodegradation within 14 days. (**a**) FDA hydrolytic activity of 0.01 g/L crude oil. (**b**) FDA hydrolytic activity of 10.00 g/L crude oil.

Figure 2 shows that the growth rates of L2A and L3A slowed down in the later period, whereas those of L2B and L3B continued to increase. *C. vulgaris* LH-1 can efficiently fix CO_2_ and provide a carbon source for bacteria and fungi. In addition, *C. vulgaris* LH-1 can metabolize organic matter. Among the samples, H3B maintained a steady growth trend. However, H2A, H2B, and H3A had a longer delay period, and the biological activity of H2B was higher at this stage. Without added nutrients, H1A and H1B entered the logarithmic growth period, in which the slow growth period was longer. A previous study reported that adding crude-oil-degrading bacteria to endogenous seawater bacteria could improve crude oil degradation and biological activity to a certain extent. This result is similar to our research results. The addition of *C. vulgaris* LH-1 can increase the biological activity (i.e., biomass) of endogenous microorganisms. In contrast, although an increase in nutrients can also increase the biomass to some extent, *C. vulgaris* LH-1 as a bioactive supplement agent is more friendly to other microorganisms.

### 3.3. OTU Cluster Analysis

As shown in Table 1, 1,065,329 archaeal sequences from 13 samples were obtained by 16S high-throughput sequencing after 14 days of culture. H2B had the fewest sequences (44,009), and L2b had the most sequences (133,024). A total of 27 phyla, 57 classes, 108 orders, 226 families, 686 genera, and 5273 OTUs were detected in the taxa.

**Figure 3:**
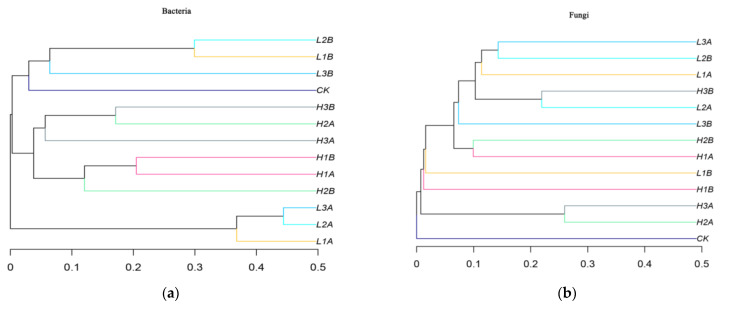
OTU cluster tree of bacteria and fungi. (**a**) Bacterial Level. (**b**) Fungi Level.

A total of 1,309,870 original fungal sequences were obtained from 13 samples by ITS high-throughput amplicon sequencing. L1B had the fewest sequences (37,333), whereas L1A had the most sequences (16,156). A total of 11 phyla, 29 classes, 64 orders, 127 families, 167 genera, and 1869 OTUs were detected in the taxa. The cluster tree diagram of the bacterial samples (Figure 3a) shows that in the seawater group containing low-concentration crude oil, L2A, and L3A were close to each other; L1B and L2B were close to each other; and CK was close to L1B, L2B, and L3B. In addition, the cluster of L1B, L2B, and L3B was far from the cluster of L1A, L2A, and L3A. In the seawater group with high crude oil concentration, H1a was close to H1B, H2A was close to H3B, and H2A was far from H2B. Figure 3b shows the cluster tree diagram of the fungal samples.CK was far from all other samples. The similarity between H3a and H2A was the highest, followed by that between L2A and H3B, between L3A and L2B, and between H1A and H2B.

### 3.4. Changes in Bacterial and Fungal Diversity under the Influence of Chlorella

Table 2 shows the alpha diversity index of bacteria and fungi in the 13 samples. Among the bacteria, L3A had the most sequences (120,588 sequences), whereas H2B had the fewest sequences (20,090 sequences). H2A and H2B had the most (1226) and fewest OTUs (571), respectively. Comparison of the species richness indices, Ace, and Chao1, revealed that the addition of *C. vulgaris* LH-1 increased the bacterial and fungal richness under the following conditions: high crude oil concentration with high or low nutrient salt concentration and low crude oil concentration with high nutrient salt concentration. However, it decreased the bacterial richness under other conditions. The addition of *C. vulgaris* LH-1 increased the abundance of bacteria in low crude oil concentrations without nutrient addition, but the opposite was true for high crude oil concentrations. In addition, the bacterial richness of *C. vulgaris* LH-1 was lower than the blank value because of its low nutrient concentration. The addition of *C. vulgaris* LH-1 increased bacterial diversity, except in environments with low crude oil and nutrient concentrations.

Among fungi, H3A had the most sequences (116,515), whereas the L1B condition had the fewest sequences (62,455). CK had the most OTUs (400), whereas L3A had the fewest OTUs (148). The abundance of all fungi affected by crude oil was lower than that in the blank level. Similar to bacterial richness, the addition of *C. vulgaris* LH-1 decreased fungal richness under low nutrient concentrations but increased the fungal richness under high nutrient concentrations.

From the perspective of diversity, the Shannon index analysis showed that the addition of *C. vulgaris* LH-1 increased fungal diversity after supplementation with low and high nutrient concentrations but decreased the fungal diversity without nutrient addition. Compared with the blank, adding *C. vulgaris* LH-1 increased fungal diversity in seawater with low-concentration crude oil and added nutrients but decreased the fungal diversity in other conditions. The coverage indices of all the samples were above 99%, which indicates that the sequencing depth was reasonable [26].

### 3.5. Effects of Chlorella on Bacterial Community Composition and Diversity

**Figure 4:**
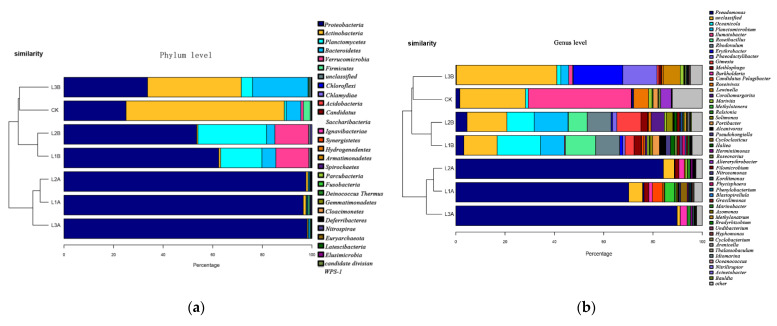
Effects of *C. vulgaris* LH-1 supplementation on bacterial community in seawater with 0.01 g/L crude oil and different nutrient concentrations. (**a**) Phylum Level. (**b**) Genus Level.

The bacterial community composition of seawater with crude oil under the influence of *C. vulgaris* LH-1 is shown in Figure 4. At the phylum level, the dominant phylum in CK was Actinobacteria, followed by Proteobacteria and Bacteroidetes. Compared with CK, the addition of *C. vulgaris* LH-1 induced regular changes, and the abundance of Proteobacteria decreased [27] considerably with increased nutrient concentration (*p* < 0.05). The relative abundance of Actinomycetes decreased in L3B compared with the blank and was extremely low in L1B and L2B. No remarkable differences in the relative abundance of *Bacteroidetes* were found between CK and L1B, but the abundance of Bacteroidetes decreased in L2B and increased in L3B. The relative abundances of Verrucomicrobiota were greater in L1B and L2B than in CK but had no substantial difference between L1B and L2B. The addition of *C. vulgaris* LH-1 decreased the relative abundance of Proteobacteria and increased the relative abundance of Phylloidetes compared with the absence of *C. vulgaris* LH-1.

At the genus level, the microbial abundance in CK was similar to that in L1B, L2B, and L3B, which were added with *C. vulgaris* LH-1. The dominant genera in CK were *Ilumatobacter* and unclassified *Acidimicrobiales*. L1B and L2B had the same main bacterial genera, namely *Oceanicola*, *Roseibacillus*, *Rhodovulum*, and *Planctomicrobium*. The dominant genera in L3B were unclassified Microbacterium, *Erythrobacter*, and *Phaeodactylibacter*.

**Figure 5:**
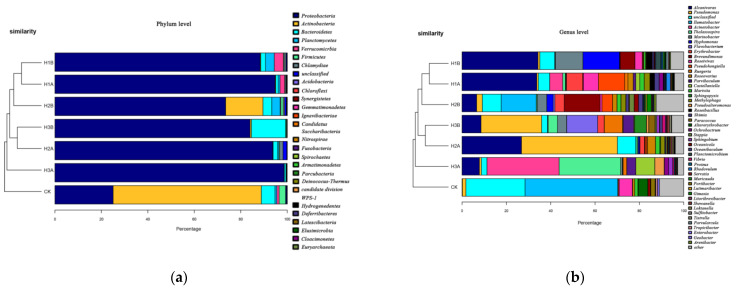
Effects of *C. vulgaris* LH-1 supplementation on bacterial community in seawater containing 10.00 g/L crude oil and different nutrient concentrations. (**a**) Phylum Level. (**b**) Genus Level.

The bacterial community composition in seawater with crude oil under the influence of *C. vulgaris* LH-1 is shown in Figure 5. In the sample cluster tree, CK was the farthest away from the other samples, and H1B was closer to H1A. The same trend was observed in the low-concentration crude oil at the gate level. Proteobacteria remained the dominant phylum under all conditions, and its abundance was lower under the influence of *C. vulgaris* LH-1 than without *C. vulgaris* LH-1. Except for the blank, the relative abundance of Actinomycetes was the highest in H2B. The abundance of *Microflora verrucosa* was higher in the environment without nutrient supplementation than in other environments but was very low in the environment with low and high nutrient supplementation. The addition of Bacteroidetes and *C. vulgaris* LH-1 also induced regular changes. *C. vulgaris* LH-1 promoted the abundance of Bacteroidetes under the influence of high crude oil concentration.

The dominant genera in H1B were *Alkanophagus*, *Rhizomonas*, and *Marinobacter*, but the abundances of these three genera were extremely low in CK. Previous studies showed that bacteria in genus *Filomonas* play a major role in the stress caused by pristane and PAHs [28].The dominant genera in H2B were *Brevundimonas*, *Ilumatobacter*, and unclassified bacteria. The dominant genera in H3B were *Pseudomonas*, *Flavobacterium*, *Alcanivorax*, and *Ruegeria*.

In the seawater group containing 0.01 g/L crude oil, some bacterial genera were affected by *C. vulgaris* LH-1, as shown in Table 3. Under the same nutritional conditions, the relative abundances of *Pseudomonas*, *Burkholderia*, *Ralstonia*, *Cladomonas*, and *Aspergillus* decreased under the influence of *C. vulgaris* LH-1. In comparison, the abundances of marine fungi, *Rhodococcus*, *Methylophaga*, *Planctomicrobium*, *Gimesia*, *Phaeodactylibacter*, and unclassified *Hortaea* increased with *C. vulgaris* LH-1. In addition, *Planctomicrobium* and *Gimesia* did not exist in the absence of *C. vulgaris* LH-1. 

### 3.6. Effects of Chlorella on Fungal Community Composition and Diversity

**Figure 6:**
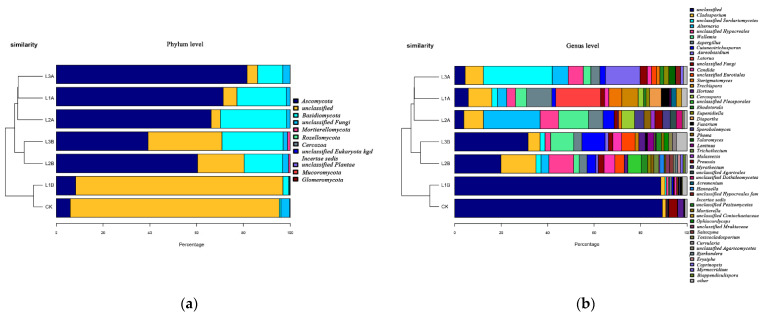
Effects of *C. vulgaris* LH-1 supplementation on fungal communities in seawater with 0.01 g/L crude oil and different nutrient concentrations. (**a**) Phylum Level. (**b**) Genus Level.

The influence of *C. vulgaris* LH-1 on the fungal community of seawater with 0.01 g/L crude oil and different nutrient concentrations is shown in Figure 6. At the phylum level, L3A, L1A, and L2A were relatively close to each other in the sample cluster tree diagram, whereas CK and L1B had a high similarity. The dominant phylum in CK was unclassified fungi (possibly eukaryotic or unidentifiable), followed by Ascomycota. The vantage gate in L1B was also unclassified. The abundance of unclassified fungi in L2B decreased, but the abundances of Ascomyces and Basidiomycota increased. L3B had the same main phyla as L2B. Compared with CK, L3B had decreased abundance of unclassified fungi and increased abundance of Ascomyces and Basidiomycota. At the inoculum level, the addition of *C. vulgaris* LH-1 decreased the abundance of Ascomycetes but increased the abundance of unclassified fungi.

The most dominant genus in CK was unclassified, followed by *Hortaea* and *Cladosporium*. The most dominant genus in L1B was also unclassified, and the only genera with relative abundances greater than 1% were *Cladoides* and *Aspergillus*. Moreover, L2B had a higher diversity index than L1B, and the top five in relative abundance were unclassified, *Unclassified_Hypocreales*, *Unclassified_Pleosporales*, and *Candida*. The dominant genera in L3B were unclassified, *Cutaneotrichosporon*, *Wallemia*, *Unclassified_Eurotiales*, and *Cladoides*. The genera in L2B and L3B differed greatly compared with those in CK.

**Figure 7:**
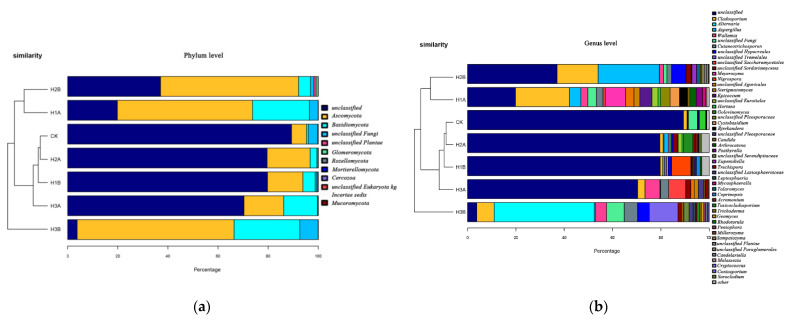
Effects of *C. vulgaris* LH-1 addition on fungal communities in seawater containing 10.00 g/L crude oil and different nutrient concentrations. (**a**) Phylum Level. (**b**) Genus Level.

The effects of the addition of *C. vulgaris* LH-1 on the fungal community in seawater with 10.00 g/L crude and different nutrient concentrations are shown in Figure 7. “Unclassified” was the dominant phylum in H1B; the abundance of “Unclassified” in H1B was greater than that in H2B but less than that in H3B. The main phyla in H1B were Ascomycetes and Basidiomycetes. Ascomycetes was the dominant phylum in H2B and H3B, and Basidiomycetes was the dominant phylum in H2B and H3B. In the sample cluster tree, CK was close to H2A, whereas H3B was the farthest from other conditional diversity. At the genus level, “Unclassified” was low in abundance in H3B but was dominant under other conditions. *Nigrospoa*, the dominant genus in H1B, was not detected under other conditions. The main genera in H2B were *Aspergillus* and *Blastomyces*. The dominant genera in H3B included unclassified *Agaricata*, unclassified fungi, and *Cladophyllus*.

### 3.7. KEGG Statistics and Difference Analysis

**Figure 8:**
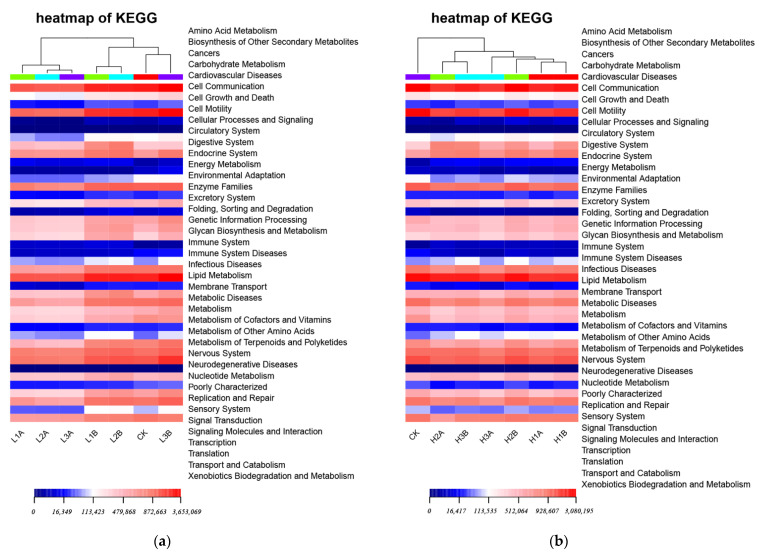
Heat map of KEGG secondary function abundance. (**a**) KEGG secondary function abundance heat map of 0.01 g/L crude oil. (**b**) KEGG secondary function abundance heat map of 10.00 g/L crude oil.

PICRUSt predicted 328 pathways based on 16S sequences, including primary KEGG pathways, as shown in Figure 8 in the heat map of secondary functional abundance based on KEGG. In the crude oil seawater group, the top 10 pathways in terms of average abundances included membrane transport, amino acid metabolism, carbohydrate metabolism, replication and repair, energy metabolism, poorly categorized functions, and translation. In addition, the differences in the function of co-factors and lipid metabolism in the sample trees were consistent with the differences in bacterial levels. Amino acid metabolism, carbohydrate metabolism, fatty acid metabolism, and sugar metabolism, as well as secondary metabolites in the metabolism and other metabolic pathways, were high because adding *C. vulgaris* LH-1 increased metabolic abundance. *C. vulgaris* LH-1 increased certain metabolic substances to provide nutrients for the bacteria, improved the activity of the bacteria, and improved the crude oil degradation efficiency.

## 4. Discussion

The crude oil degradation efficiency in seawater polluted by crude oil was 26.60%, 46.45%, and 60.69% without nutrient addition, with low nutrient addition, and with high nutrient addition, respectively. The introduction of *C. vulgaris* LH-1 increased the degradation efficiency to 29.55%, 66.15%, and 70.13%, representing an increase of 11.09%, 42.41%, and 15.60%, without nutrient addition, with low nutrient addition, and with high nutrient addition, respectively. The bacterial–algal system improved the degradation effect of aliphatic and aromatic oils [29]. In seawater containing low-concentration crude oil, the oil degradation efficiency was close to 100% after the addition of nutrients, and the degradation rate of *C. vulgaris* LH-1 was remarkably accelerated after the enhancement. Bacteria, fungi, and *microalgae* in the environment are able to remove hydrocarbon pollutants [11,12,30]. The complex community composed of bacteria, fungi, and *microalgae* can biodegrade hydrocarbons [31,32]. Among all microorganisms, bacteria are the main degrading organisms and the most active factors in the degradation of petroleum pollutants [33,34]. The combination of algae and bacteria produces many beneficial effects. Microalgae are responsible for photosynthesis and can provide oxygen for bacteria to absorb and degrade the CO_2_ released by organic pollutants. Therefore, the enhancement of *C. vulgaris* LH-1 increased the crude oil degradation efficiency.

*C. vulgaris* LH-1 enhanced the remediation of seawater containing 10.00 g/L crude oil. The dominant bacteria in the crude-oil-containing seawater without additional nutrients were *Alcanivorax* and *Rhizomonas*. Bacteria in *Filomonas* play a major role in the stress of pristane and PAHs [28]. The dominant species in seawater with 0.02 g/L nutrient restoration was *Brevunmdimonas*, whereas the dominant species in seawater with 2.00 g/L nutrient restoration was *Halomonas*. The dominant genera were different under the three different nutritional conditions, but all had the highest abundance of *Alcanivorax*. Alkylophagous fungi have strong hydrocarbon degradation ability and can use alkanes and various alkyl benzene derivatives as carbon and energy sources. Since the discovery of the alkyl-eating bacteria, all isolated strains have been reported to degrade aliphatic compounds [35]. In the present study, the abundance of *Alcanivorax* was relatively low in seawater containing low crude oil concentrations, which indicates that high hydrocarbon concentrations can stimulate the growth of *Alcanivorax*. In contrast, *Methylotrophs* were found only in seawater with low crude oil concentrations and were not detected in seawater with high crude oil concentrations. *Methylotrophs* can grow in environments where *n*-acetane is the only carbon source and energy source, proving that they have the ability to degrade hydrocarbons [36].

The enhancement caused by *C. vulgaris* LH-1 changed the community of bacteria and fungi in oil-polluted seawater; fungi can be also co-cultured with other bacteria and fungi to enhance hydrocarbon biodegradation [37]. The results of high-fluence sequencing showed that in the seawater group containing low crude oil concentrations, the main bacterial genera were marine bacteria, such as *Roseibacillus* and *Rhodonococcus*, regardless of nutrient salt addition. The main bacterial genera added with high nutrient concentration were unclassified Micrococcaceae, *Erythrobacter*, and *Phaeodactylibacter*. In addition, the abundance of Marine bacteria, *Rhodococcus*, and *Gibberella* increased with the addition of *C. vulgaris* LH-1, whereas the abundance of *Pseudomonas*, *Cladiomyces*, and *Aspergillus* decreased. Under the influence of *C. vulgaris* LH-1, the abundance of *Cladophyllus* and *Aspergillus* decreased, whereas the abundance of *Hortaea* increased. The addition of chloreous algae at high crude oil concentrations increased the abundance of unclassified *Hypocreales*, which were isolated from a variety of tar spheres and are able to rapidly mineralize benzopyrene [38]. *Cryozoa* is a common oil-degrading bacterium. Its abundance was very low in the seawater environment with 2.00 g/L nutrient added, but its abundance was high at a lower nutrient concentration. Some studies found that the *Erythrobacter* isolated from *C. vulgaris* LH-1-treated seawater has 95% homology with genus *Cryozoa* [39]. This may be the reason for the increase in the abundance of *Cryozoa* under the influence of *C. vulgaris* LH-1. *Erythrobacter* is a genus of the Erythrobacter family. Many PAH-degrading strains have been isolated from marine sediments. Zhou [40] used co-metabolism technology to degrade coking wastewater in a reactor and found *Planctomicrobium* as the main genus in the system containing glucose and a small amount of urea. Studies showed that *C. vulgaris* LH-1 can metabolize polysaccharides, proteins, and other substances and provide available carbon sources for *Planctomicrobium* to improve its abundance. *Pseudomonas* has a strong ability to degrade PAHs and can also produce biosurfactants; therefore, it has been widely used in oil spill treatment [41,42].

## 5. Conclusions

In this study, the degradation efficiency of crude oil was improved by the addition of *C. vulgaris* LH-1, regardless of the addition of low- or high-concentration nutrients in the seawater polluted by crude oil. The degradation efficiencies in seawater containing high crude oil concentrations without nutrient salt, with low-nutrient salt, and with high-nutrient salt were 26.60%, 46.45%, and 60.69%, respectively. The introduction of *Chlorella* increased the degradation efficiencies to 29.55%, 66.15%, and 70.13%, representing an increase of 11.09%, 42.41%, and 15.60%, without nutrient salt, with low-nutrient salt, and with high-nutrient salt, respectively. The oil degradation efficiency in seawater containing low-concentration crude oil was close to 100% after the addition of nutrients, and the degradation rate of *C. vulgaris* LH-1 was considerably accelerated after the enhancement. High-throughput sequencing results showed that *C. vulgaris* LH-1 intensively repaired seawater containing 0.01 g/L crude oil. The addition of 0.02 g/L nutrient changed the main species to marine species, such as *Roseibacillus*. The addition of 2.00 g/L nutrient in the repair of oil-containing seawater changed the main species to unclassified microbacteria, *E. coli*, and *Phaeodactylibacter*. Moreover, the addition of *C. vulgaris* LH-1 increased the abundance of marine bacteria, *Rhodococcus*, and *Gibberella* and decreased the abundance of *Pseudomonas*, *Cladiomyces*, and *Aspergillus*. The enhanced remediation of *C. vulgaris* LH-1 contained 10.00 g/L crude oil seawater group, and the dominant bacteria in the seawater without additional nutrients were *Alcanivorax borkumensis* and *Rhizomonas*. The addition of 0.02 g/L nutrients changed the dominant genus to *Brevunmdimonas*, and the addition of 2.00 g/L nutrients changed the dominant genus to *Halomonas*.

## Figures and Tables

**Table 1:** Bacteria and fungi information statistics.

Classification	Sample	Barcode	Raw_num	Mean_len	Clean_num	Mean_len
Bacteria	CK	GTCCCA	54,361	447.82	54,241	405.60
	L1A	ATCGCA	86,397	465.70	86,238	424.52
	L1B	TTACGA	113,931	453.07	113,690	411.30
	L2A	TGTTAT	92,096	467.21	91,959	426.35
	L2B	GCCATC	133,024	451.07	132,736	409.36
	L3A	TGTGTT	126,295	467.90	126,121	427.23
	L3B	TAGGAC	68,698	451.40	68,644	410.60
	H1A	TGGACG	45,333	459.60	45,296	418.81
	H1B	CGATGT	59,305	458.41	59,250	417.59
	H2A	TCCTGT	113,531	464.18	113,409	422.99
	H2B	GAAGGC	44,009	449.19	43,957	407.94
	H3A	ATGTCA	44,843	458.82	44,805	417.50
	H3B	CGGTTA	83,506	456.44	83,394	415.26
Fungi	CK	TACGACA	90,066	326.10	89,182	283.14
	L1A	TGTGCTA	161,561	162.87	75,719	236.57
	L1B	TCACTCG	67,333	344.81	66,047	305.03
	L2A	TACTCTG	94,398	235.68	77,000	232.07
	L2B	TGGACTC	99,821	284.55	98,355	243.28
	L3A	TCTGTAG	95,305	265.14	88,416	237.09
	L3B	TACACTC	89,859	251.21	81,584	224.43
	H1A	TGATCGC	84,263	271.11	80,680	235.59
	H1B	TCGTCAT	116,317	263.11	80,979	288.47
	H2A	TATCAGC	97,088	247.41	89,636	217.09
	H2B	TGCGACG	85,628	265.37	77,701	236.30
	H3A	ATACAGA	134,335	206.03	116,955	178.19
	H3B	ATGTCTC	93,896	264.41	90,499	227.13

**Table 2:** Sequence analysis of bacterial and fungal samples.

Classification	Sample	Seq_num	OTU	Shannon	ACE	Chao1	Coverage	Simpson
Bacteria	CK	37,907	907	2.9526	1025.1650	958.0203	0.9954	0.2005
	H1A	40,297	771	3.2891	1185.5860	1086.3910	0.9919	0.1139
	H1B	50,637	920	3.1865	1302.4010	1144.3850	0.9935	0.1328
	H2A	93,149	1226	2.5940	1594.5820	1461.0970	0.9958	0.2437
	H2B	20,090	571	3.8727	901.8986	808.0522	0.9884	0.0624
	H3A	36,790	574	2.3597	861.5187	760.2338	0.9935	0.1981
	H3B	57,011	881	3.2164	1270.6230	1127.2820	0.9940	0.1033
	L1A	80,987	888	2.3545	1183.1880	1135.9590	0.9964	0.2548
	L1B	75,251	897	3.7709	1113.4030	1038.3030	0.9969	0.0656
	L2A	86,414	854	1.7350	1109.2600	1045.9510	0.9969	0.3662
	L2B	63,471	703	3.7776	852.4927	811.2891	0.9974	0.0511
	L3A	120,588	915	1.4163	1173.5610	1069.3960	0.9978	0.4090
	L3B	57,488	944	2.8666	1303.8270	1195.3190	0.9943	0.1677
Fungi	CK	87,785	400	3.2318	416.1471	405.1786	0.9997	0.0898
	H1A	80,081	219	3.2326	563.8095	333.4872	0.9988	0.0588
	H1B	79,488	354	3.1260	370.2629	357.9545	0.9996	0.0946
	H2A	88,090	242	2.3100	290.8538	270.5577	0.9994	0.2681
	H2B	77,243	173	2.9895	322.7502	241.0556	0.9994	0.0969
	H3A	116,515	205	2.0583	260.2814	238.5490	0.9995	0.3007
	H3B	89,889	173	2.4210	246.1856	224.4839	0.9994	0.2019
	L1A	75,342	152	3.0662	352.5439	219.5357	0.9992	0.0722
	L1B	62,445	325	1.8302	359.2254	336.0588	0.9992	0.3602
	L2A	76,472	198	2.8612	303.8780	285.5676	0.9989	0.0950
	L2B	97,354	184	3.4065	229.1768	213.4000	0.9995	0.0540
	L3A	87,757	148	2.7871	228.3164	199.2069	0.9994	0.1173
	L3B	80,723	202	3.4413	274.5173	242.5263	0.9993	0.0579

**Table 3:** Bacteria affected by *C. vulgaris* LH-1 at low crude oil concentration.

Phylum	Genus	Abundance Ratio %						
		CK	L1A	L1B	L2A	L2B	L3A	L3B
Proteobacteria	*Oceanicola*	1.14	0.09	17.59	0.02	11.04	0.01	1.62
	*Rhodovulum*	0.13	0	9.74	0.01	9.71	0	0.34
	*Erythrobacter*	0.42	0.36	1.27	0	0.54	0.02	20.08
	*Methylophaga*	0	1.97	2.89	1.58	2.77	0.01	0.95
	*Pseudomonas*	1.59	70.12	3.27	84.21	4.57	89.77	0.23
	*Burkholderia*	0.04	1.42	0.1	2.2	0.17	2.73	0.01
	*Ralstonia*	0.01	0.77	0.06	1.06	0.08	1.41	0.01
Planctomycetes	*Planctomicrobium*	0.02	0	9.72	0	13.78	0	3.17
	*Gimesia*	0.03	0	3.63	0	9.74	0	0.71
Bacteroidetes	*Phaeodactylibacter*	0.27	0.02	0.92	0.04	1.78	0.02	13.88

## Data Availability

All data, models, and code generated or used during the study appear in the submitted article. The raw/processed data required to reproduce these findings cannot be shared at this time, as the data also form part of an ongoing study.
